# From play to profession: understanding the professionalization of e-sports athletes from a civilizing process perspective

**DOI:** 10.3389/fpsyg.2026.1912932

**Published:** 2026-09-23

**Authors:** Wei-jie Hu, Cong-hui He

**Affiliations:** 1 School of Economics and Management, Shanghai University of Sport, Shanghai, China; 2 School of Physical Education, Shanghai University of Sport, Shanghai, China

**Keywords:** career development process, civilization theory, elias, e-sports, e-sports athletes

## Abstract

**Introduction:**

Despite the rapid expansion of the e-sports industry in terms of economic scale, tournament systems, and professional demand, the issues of identity transformation, bodily discipline, emotional management, and their associated alienation during the professionalization of e-sports athletes remain insufficiently discussed. Grounded in Elias’s theory of the civilizing process, this study aims to explore the professionalization process of e-sports athletes as they transition from amateur players to professional competitors, as well as the relationships among social constraints, bodily practices, and emotional internalization within the digital competitive arena.

**Methods:**

Using historical analysis, case study, and semi-structured interviews, this study takes current and former professional e-sports athletes in China as empirical subjects.

**Results:**

The study reveals three civilizing mechanisms in the professionalization process of e-sports athletes, namely the institutionalization of identity, the rationalization of bodily discipline, and the internalization of emotional management. The findings indicate that the professional development of e-sports athletes is driven by interdependency networks composed of capital, leagues, clubs, data platforms, and fan communities, with digital technology serving as a mediator to facilitate the transformation from external constraints to individual self-constraint.

**Discussion:**

The study points out that the professionalization of e-sports also faces dilemmas such as the alienation of bodily discipline, imbalance in power dependency, and lack of health protection. This study proposes that a sustainable support mechanism for the professional development of e-sports athletes should be improved from two aspects, which are a dual-dimensional health protection system encompassing both physical and psychological aspects, and the enhancement of individual subjectivity.

## 1 Introduction

e-sports is generally understood as organized competitive activity enabled by information and communication technologies. Its essence lies not in mere gaming and entertainment, but in digital competition grounded in operational proficiency, tactical understanding, team coordination, and psychological control (Reitman et al., 2020; Musick et al., 2021). With the evolution of digital technologies, the expansion of platform economies, and the upgrading of global cultural consumption, e-sports has gradually transformed from arcade-based entertainment in the 1980s into a significant component of the global digital cultural industry. According to Newzoo’s 2024 Global Games Market Report, the global games market has reached a scale of hundreds of billions of US dollars and possesses a vast user base, providing a critical industrial environment for the development of e-sports tournaments, club operations, platform dissemination, and fan economies (Newzoo, 2024).

In this process, e-sports athletes have ceased to be mere game participants and have become key actors connecting tournament systems, club management, platform communication, commercial capital, and fan communities. The rapid development of China’s e-sports industry has further propelled the legitimation and institutionalization of the professional identity of e-sports athletes. From an institutional perspective, the National Electronic Sports Athlete Registration and Transfer Management Measures (Trial), issued by the All-China Sports Federation, provides a regulatory framework for the registration management, tournament participation, and transfer order of e-sports athletes, establishing a crucial foundation for the recognition of their professional identity (All-China Sports Federation, 2018). The inclusion of e-sports as an official competitive event in the 2022 Hangzhou Asian Games further reinforced the competitive and professional attributes of e-sports through the authority of an international multi-sport event. Thus, the professionalization of e-sports athletes is not driven solely by the improvement of individual competitive ability, but is the outcome of the interplay among national regulations, tournament rules, club management, platform technologies, and market capital.

Existing research has examined e-sports from multiple perspectives. Relevant contributions primarily include conceptual debates and historical evolution regarding whether e-sports qualifies as a sport (Jenny et al., 2017; Hallmann and Giel, 2018; Parry, 2019); institutional development, business ecosystems, and governance structures in the process of e-sports industrialization (Scholz, 2019; Peng et al., 2020; Holden et al., 2017); studies on e-sports media communication, audience culture, and platform interaction (Hamari and Sjöblom, 2017; Pizzo et al., 2018; Qian et al., 2020); research on the professional identity construction and digital labor relations of e-sports participants (Seo, 2016a; Brock, 2017; Johnson and Woodcock, 2021); and studies on training discipline, occupational health, and psychological stress among e-sports athletes (Kari and Karhulahti, 2016; DiFrancisco-Donoghue et al., 2019; Poulus et al., 2020). Regarding the sporting status of e-sports, Kane and Spradley argue that e-sports possess several core features of sport, including institutionalized competition, specialized skills, formal rules, and organized tournaments (Kane and Spradley, 2017). Molina et al., however, suggest that the “sportization” of e-sports does not make e-sports identical to traditional sport. Instead, the two fields are becoming increasingly connected through a continuing process of interaction (Molina et al., 2025). e-sports also differ from traditional sports in their governance structure. In most conventional sports, rules are established by independent governing bodies. In e-sports, game developers often control competition rules, software updates, and tournament authorization. This creates a governance model shaped by commercial ownership and intellectual property rights. Moreover, titles such as Counter-Strike, DOTA 2, Honor of Kings, and Overwatch contain varying degrees of combat and simulated violence. The IOC has stated that some electronic games are incompatible with Olympic values and are therefore excluded from cooperation. The integration of e-sports into the Olympic Movement thus depends not only on their competitive and organizational characteristics, but also on the compatibility of their content with Olympic values (International Olympic Committee, 2018).

Although existing studies have contributed to understanding e-sports industrial structure, governance, and cultural communication, several limitations remain. First, previous research has primarily focused on macro-level issues such as industry development, policy regulation, platform governance, and cultural practices (Seo, 2016a; Taylor, 2012; Funk et al., 2018). Second, existing research has not fully explained how tournament systems, club management, platform data, and public discourse reshape athletes’ identities, bodies, and emotions (Seo, 2016b). Third, insufficient attention has been paid to the transformation of play into work, the decline of athlete autonomy, and unequal relations of dependence during professionalization. Drawing on Blanchard and Cheska’s distinction between play and work, e-sports professionalization may provide athletes with income, occupational identity, and social recognition. At the same time, it may transform voluntary and recreational gaming into occupational labor governed by training schedules,contractual obligations, performance targets, and commercial activities (Blanchard and Cheska, 1985). Professionalization therefore involves more than regulation and institutionalization. It may also lead to the instrumentalization of athletes’ bodies, the suppression of emotional expression, and the erosion of occupational autonomy.

This study adopts Norbert Elias’s theory of the civilizing process as its core analytical framework. Elias does not regard civilization as a linear form of progress. Instead, he conceptualizes it as a long-term historical process in which networks of interdependence expand and external constraints are gradually transformed into self-restraint. As the division of labor deepens and chains of interdependence lengthen, individual conduct becomes increasingly shaped by institutional norms, the expectations of others, and social evaluation (Elias, 2000). This process fosters greater control over bodily conduct, emotional expression, and impulses. In the sociology of sport, Elias and his collaborators further conceptualize modern sport as a civilizing arena in which social regulation, emotional control, and bodily restraint are closely intertwined. Their work highlights the historical relationship between external rules and individual self-restraint (Elias and Dunning, 1986). The theory distinguishes between two main dimensions: sociogenesis and psychogenesis. Sociogenesis concerns changes in the division of labor, power relations, institutional norms, and systems of interdependence. Psychogenesis focuses on the formation of bodily habits, emotional regulation, and self-control (Mennell, 1998).

In the professionalization of e-sports, athletes become embedded in networks of interdependence involving clubs, tournament leagues, game developers, commercial actors, streaming platforms, and fan communities. Contracts, competition rules, training metrics, platform data, and public discourse regulate their professional conduct from the outside. At the same time, intensive training, frequent competition, and routine livestreaming continuously reshape their daily routines, bodily practices, and emotional expression (Lin and Zhao, 2020). To maintain competitive opportunities, contractual relationships, and professional reputations, athletes increasingly regulate their own behavior. External managerial requirements are thus gradually internalized as professional norms. Digital platforms, data systems, and communication media further increase the visibility of rules and evaluation. As a result, e-sports professionalization acquires distinct platform-mediated and data-driven characteristics.

Building on this framework, the study develops two analytical concepts: the civilizing pathway of professionalization and disciplinary internalization, which explain how amateur players become professional athletes and how external institutional constraints are transformed into internalized professional dispositions.

The overall aim of this study is to examine the professionalization process of e-sports athletes through Elias’s theory of the civilizing process, with particular attention to how external social constraints are gradually transformed into internalized professional norms and self-regulatory mechanisms. Specifically, this study pursues three objectives: first, to explore how professional identities are constructed through institutionalization; second, to analyze how bodily discipline is rationalized through training systems and performance evaluation; and third, to examine how emotional regulation becomes internalized as part of professional competence.

Through these objectives, this study further investigates the dual nature of e-sports professionalization. While the civilizing process promotes professional development, stabilizes competitive practices, and facilitates the formation of professional identities, it may also generate new forms of control, dependency, and alienation. Therefore, e-sports professionalization is understood not only as a process of institutional development but also as a field of tension between civilizing advancement and individual constraint.

## 2 Method

### 2.1 Research design

This study is situated within the interpretivist research paradigm, approaching the research topic from the subjective experiences of individual athletes. It emphasizes that reality is not externally given, but is continuously understood, interpreted, and constructed within specific institutional environments, professional relationships, and interactive contexts (Creswell and Poth, 2017). Accordingly, this study explores the logic underlying the transformation from amateur players to professional e-sports athletes under the combined constraints of industry regulations, tournament rules, club management, platform data, and fan discourse, while attending to both the macro-level historical evolution of professionalization and athletes’ micro-level professional experiences.

This study employs the literature review method, case analysis method, and semi-structured interviews. Documentary and institutional materials are used to examine the historical evolution, institutional environment, and organizational relationships involved in the professionalization of e-sports athletes. Interview data are used to capture athletes’ specific experiences of professional identity, bodily practices, and emotional management. By combining macro-level institutional materials with micro-level experiential data, this study examines how external institutional constraints shape athletes’ professional practices and are gradually transformed into forms of self-management and professional norms.

### 2.2 Data sources and participants

Documentary and institutional materials include academic publications, industry reports, tournament rules, industrial policies, and club documents. These are used to trace the developmental trajectory of e-sports from informal gaming activities to professionalized and institutionalized competition. Documentary and institutional texts help researchers identify the relational structures among policies, organizations, and actors in the absence of complete quantitative data (Bowen, 2009). The case analysis method is suitable for analyzing the complex interactions among institutions, organizations, and individual practices within specific industry contexts (Yin, 2009).

For primary data collection, this study combines purposive sampling and snowball sampling to select 15 current or former professional e-sports athletes in China for online semi-structured interviews. Purposive sampling is used to select respondents with typical experiences and rich information relevant to the research questions. Snowball sampling is suitable for accessing a research field that is relatively closed and characterized by strong member networks (Patton, 2002; Noy, 2008).

All respondents have professional experience within the e-sports professional system in China, and their career experiences occurred primarily within Chinese clubs, tournaments, and platform environments. Respondents are primarily players of mobile MOBA games, with additional players of PC-based MOBA and FPS games included to enhance sample diversity in terms of game genre and career experience. The main games represented include Honor of Kings, DOTA 2, Counter-Strike: Global Offensive, and Overwatch.

Data saturation was assessed continuously as data collection and preliminary analysis proceeded in parallel. The researchers compared the professional accounts of athletes from mobile MOBA, PC-based MOBA, and FPS games. When subsequent interviews generated no new codes or themes related to professional identity, bodily discipline, or emotional management, the data were considered saturated at the level of the overall themes. Given the uneven distribution of participants across game genres, this study does not claim that saturation was achieved independently within each genre. Instead, cross-genre comparison was used to examine the consistency of the core themes and identify differences across competitive contexts.

Among the respondents, 11 are current professional e-sports athletes, and 4 are retired professional e-sports athletes. In this study, retired professional e-sports athletes refer to individuals who had previously entered the professional e-sports system, participated in professional teams and competitions, but had subsequently withdrawn from active professional competition. The ages of respondents range from 20 to 24 years, with professional career durations of 2 to 4 years. Although retired athletes have left the professional arena, their ability to retrospectively recount their entry experiences, training regimes, club management, tournament pressure, and pre-retirement professional experiences provides valuable diachronic material for understanding the professionalization process of e-sports athletes.

The pathways through which respondents transitioned from amateur players to the professional system include three main types: progression from high-level performance to trial or youth training and then to a professional team; absorption from youth training teams into professional teams; and transition facilitated by referrals from acquaintances that attracted club interest. Basic information about the respondents is presented in Table 1.

**Table 1:** Demographics of interviewees.

Code	Game type	Primary competitive game	Age (years)	Athlete status	Transition pathway to professional	Years as professional	Interview duration (minutes)
A1	Mobile MOBA	Honor of Kings	22	Current Professional	High-level performance → trial/youth academy	4	48
A2	FPS	CS: GO	22	Current Professional	High-level performance → trial/youth academy	3	54
A3	Mobile MOBA	Honor of Kings	20	Current Professional	High-level performance → trial/youth academy	2	53
A4	Mobile MOBA	Honor of Kings	23	Current Professional	High-level performance → trial/youth academy	3	54
A5	Mobile MOBA	Honor of Kings	24	Current Professional	Youth academy → professional team recruitment	4	47
A6	Mobile MOBA	Honor of Kings	23	Current Professional	High-level performance → trial/youth academy	3	53
A7	PC MOBA	DOTA2	24	Current Professional	Youth academy → professional team recruitment	4	54
A8	PC MOBA	DOTA2	22	Current Professional	Youth academy → professional team recruitment	3	53
A9	FPS	CS: GO	23	Current Professional	Youth academy → professional team recruitment	4	46
A10	Mobile MOBA	Honor of Kings	22	Current Professional	Referral by acquaintance → club attention	3	47
A11	Mobile MOBA	Honor of Kings	21	Current Professional	High-level performance → trial/youth academy	2	47
A12	FPS	Overwatch	23	Retired Professional	Youth academy → professional team recruitment	4	47
A13	Mobile MOBA	Honor of Kings	23	Retired Professional	High-level performance → trial/youth academy	4	51
A14	Mobile MOBA	Honor of Kings	23	Retired Professional	Youth academy → professional team recruitment	3	53
A15	Mobile MOBA	Honor of Kings	23	Retired Professional	High-level performance → trial/youth academy	3	53

### 2.3 Interview procedure and research ethics

The average duration of each semi-structured interview was approximately 50 min. The interview guide was structured into three parts. The first part focused on identity, covering topics such as entry into the profession, contract and salary, external recognition, and the construction of professional identity. The second part addressed bodily discipline, concentrating on changes in daily routines after professionalization, quantified training, post-match review systems, and experiences of the body being instrumentalized. The third part explored emotional management, examining emotional regulation in situations such as in-game mistakes, livestreaming, and online public opinion, as well as the constraints imposed by external rules on emotional expression.

This study follows the ethical standards of social science research. All respondents voluntarily participated on the basis of informed consent. Respondents had the right to terminate the interview at any time or to decline to answer sensitive questions. Since the interviews were conducted online, particular attention was paid to anonymization, data confidentiality, and the protection of sensitive information (Eysenbach and Till, 2001).

Audio recordings were transcribed and anonymized with codes ranging from A1 to A15. Privacy-related and controversial content was summarized rather than directly quoted, and information that could reveal respondents’ identities was excluded. The transcripts were imported into NVivo 11 for coding.

### 2.4 Data analysis

The interview data were analyzed using the six-phase thematic analysis method proposed by Braun and Clarke (Braun and Clarke, 2006). Analysis began with repeated immersion in interview transcripts. Next, open coding captured text segments concerning professional entry, identity transformation, training practices, emotional expression, organizational management, and institutional constraints. Initial codes were subsequently clustered into candidate themes. The provisional themes were first reviewed against the full dataset. Following the review, themes were further refined, defined and formally named. Finally, coherent analytic narratives were developed. This process yielded three core dimensions: the institutionalization of professional identity, the rationalization of bodily discipline, and the internalization of emotional management.

The data analysis combined inductive coding with theoretical interpretation. Open coding was used to identify significant aspects of respondents’ professional experiences, while Elias’s theory of the civilizing process served as a sensitizing framework for theme development and interpretation. Sociogenesis was used to analyze changes in tournament systems, club management, platform governance, and networks of interdependence. Psychogenesis was used to examine changes in athletes’ bodily habits, emotional expression, and self-restraint. The transformation of external constraints into self-restraint was used to explain how tournament rules, managerial requirements, performance metrics, and public evaluation were gradually internalized into athletes’ professional conduct and self-management.

## 3 Evolutionary logic of the professionalization process of e-sports athletes

In *the Civilizing Process: Sociogenetic and Psychogenetic Investigations*, Elias argues that the civilizing process does not imply the elimination of impulses and violence; rather, it refers to the gradual regulation of such behaviors through social norms, institutional rules, and individual mechanisms of self-restraint (Funk et al., 2018). Within modern competitive systems, conflict and confrontation are not eradicated but transformed into acceptable forms of competition through regulatory frameworks and organizational institutions. As a digital competitive activity centered on confrontation and contestation, e-sports similarly embodies this civilizing logic.

Although e-sports titles such as Counter-Strike, DOTA2, Honor of Kings, and Overwatch incorporate virtual elements of shooting, elimination, and combat, these actions should not be equated with physical violence in the real world. Instead, they represent forms of competitive expression structured and constrained by game rules, technological systems, and tournament institutions. The cautious stance adopted by the IOC toward certain violent electronic games also reflects the value negotiations involved in e-sports’ transition from recreational activity to an institutionalized competitive system. Therefore, the civilizing process of e-sports does not involve the elimination of conflict itself; rather, it transforms virtual conflict into forms of competition compatible with modern competitive norms through regulatory systems, organizational standards, and sporting ethics.

This competitive normativization driven by game rules provides the institutional foundation for the formation of e-sports athletes’ professional identities. Building upon this foundation, the professionalization process of e-sports athletes further reflects Elias’s emphasis on the mutual shaping of social structures and individual psychology. As e-sports gradually shifts from recreational gaming toward an institutionalized professional system, players must continuously adapt to new social roles, organizational expectations, and behavioral norms. Through the transformation of external institutional constraints into internalized self-regulation, players gradually develop a professional subjectivity. However, this civilizing process is not a linear progression toward greater autonomy. While institutional regulation promotes professional development and stabilizes competitive practices, it may also generate new forms of control, dependency, and alienation by increasingly incorporating players’ identities, bodies, and emotions into professional systems. Specifically, this civilizing process unfolds through three interconnected dimensions: the institutionalization of identity, the rationalization of bodily discipline, and the internalization of emotional management. This process is illustrated in Figure 1.

**Figure 1:**
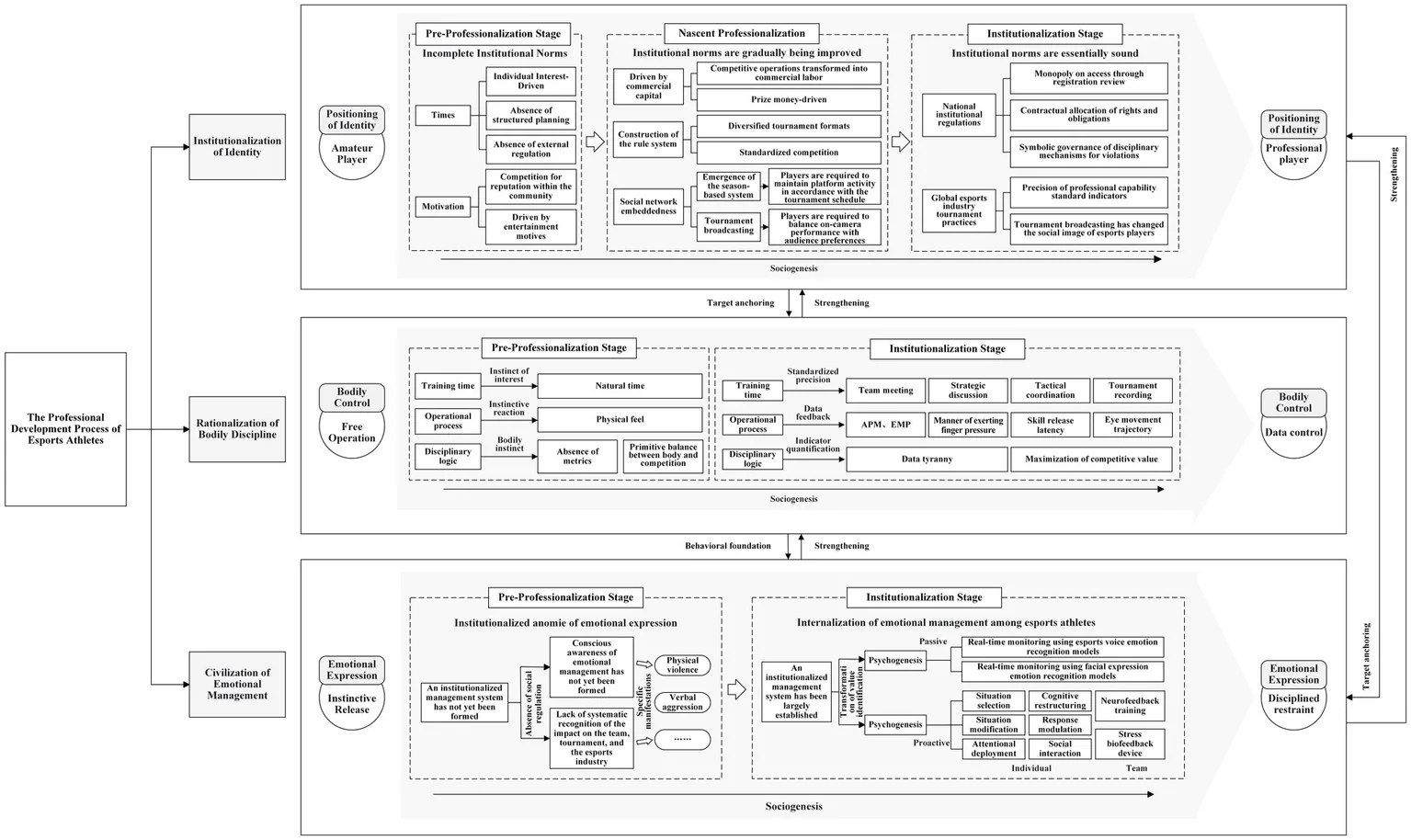
The professional development process of e-sports athletes.

### 3.1 Institutionalization of identity: from “amateur player” to “professional athlete”

Elias points out that the transformation of identity always presupposes an increasing reliance on social roles. As an individual gains resources from a particular role network, he internalizes the norms of that network into his self-identity. The identity transformation of e-sports athletes fully conforms to this principle.

#### 3.1.1 Pre-professionalization stage: “untamed” individuality and diffuse interaction

Before the 1990s, the player identity of e-sports athletes was highly salient, and individual behavior lacked systematic social control, presenting a primordial form characterized by complete autonomy in terms of time and motivation. The training and competition during this stage exhibited randomness and purity. In terms of the randomness of training duration, early arcade game players practiced entirely based on personal interest, without any fixed schedule. For example, before the 1981 California Asteroids State Championship, California player Paul Dean played Asteroids continuously for 12 h at a 7-Eleven convenience store to improve his performance; the duration and intensity of his training depended entirely on his own will, without any external constraints (Dean, 1981). This entertainment-driven form remained at a stage where emotions were expressed without restraint, characterized by the randomness of training duration and the pureness of competitive motivation.

Regarding competitive motivation, players at this time participated because they enjoyed the thrill of competing against skilled opponents, rather than viewing it as a career path. In the Pac-Man tournament held at a Putt-Putt Golf & Games entertainment center in 1981, contestant Steve Heir won with a score of 372,600 points and earned a prize of $5,500. However, his original intention for participating was simply to enjoy the process of competing against other players at the arcade (Smith, 2013; Putt Putt Golf and Games, 1981). In the 1982 Twin Galaxies National Defender Championship, 24 contestants simultaneously competed for high scores in Defender at 24 locations across North America. Their core motivation was to prove their skills within the player community, rather than to pursue professional development. The competitive goal remained at the level of gaining reputation within the community and entertainment-driven motives. At this stage, the competitive motivation of e-sports players had not yet been fully integrated into the value evaluation system of professionalization (Jones and Day, 2018).

In essence, the player identity at this stage represents a concrete manifestation of untamed individuality in the digital age within the virtual competitive arena. Although its behavioral logic still retains the spontaneity and disorder characteristic of the pre-sport stage of traditional sports, it differs from purely non-utilitarian entertainment, exhibiting a transitional feature of “interest-led, commerce-embellished,” which lays the groundwork for the value reconstruction of subsequent professionalization.

#### 3.1.2 Budding professionalization: capital intervention and the reshaping of “interest as labor”

The budding concept of the e-sports professional began with the reshaping of “interest as labor” through capital logic, a process that involved the coordinated effects of commercialization, rule system construction, and social network embeddedness.

Driven by capital, the 1990 Nintendo World Championships, sponsored by cross-industry brands such as Pepsi-Cola and Oreo, took the form of a 30-city tour with audiences of 30,000 per event. It transformed players’ competitive performances in Super Mario Bros. and Tetris into commercial labor for console promotion. The winner, Jeff Hansen, participated in a nationwide roadshow as a brand ambassador, with his gaming skills directly converted into brand communication value (Daniel, 1993).

The construction of a rule system facilitated the taming of primitive competitive disorder. In 1991, Japan’s “Gamest Cup” Street Fighter II tournament pioneered a single-elimination format with 512 players, regulating competition through drawing lots for qualification and fixed match rules (Inokawa, 2019). In 1995, DWANGO’s “Deathmatch” competition further clarified competitive rules, match duration, and criteria for determining victory, while utilizing network servers for unified management. The significance of the rule system lies not in restricting behavior, but in achieving a higher degree of social cooperation by clarifying boundaries. Players’ adherence to timing rules and scoring systems marked the beginning of the transformation from primitive competitive impulse to rational behavior.

The embedding of social networks further facilitated the transformation from external control to self-constraint. In 1997, the AMD Professional Gamers League (PGL) adopted a professional sports season system of “qualifiers – league – finals,” binding the interests of players and sponsors, and required players to maintain platform activity according to the tournament schedule to ensure commercial exposure (Li, 1997). Meanwhile, organizations such as the Cyberathlete Professional League (CPL) began to explore fixed compensation and contractual management models, forming early patterns of professional employment relationships (Miller, 1997).

At this point, player behavior had become constrained by capital logic and market demands, with primitive habits being tamed by the rule system. External constraints such as “training on time” and “complying with business contracts” began to replace instinctual interest, just as medieval knights gradually submitted to the summons of the royal court due to their dependence on the king’s land grants. The identity of players was initially tamed. Although this stage had not yet established an institutional recognition of professional identity, it laid the historical foundation for the maturation of the professional system.

#### 3.1.3 Institutionalization stage: rule system and the civilizing construction of “professional identity”

At the beginning of the 21st century, the institutionalization of the professional identity of e-sports athletes became a critical pathway for industry development. This process resulted from the combined effects of national administrative regulations and global industrial practices, and it also represents a typical manifestation of the social civilizing process in the digital age. At this stage, professional identity became embedded within an interdependent network of capital, leagues, clubs, players, data platforms, and fans.

National institutional regulations provided a legal and legitimacy foundation for professional identity. In 2006, the All-China Sports Federation successively issued the *Administrative Measures for the Registration and Exchange of National e-sports Athletes* (Trial; All-China Sports Federation, 2018; hereinafter referred to as the Registration Measures) and the *Implementation Measures for the National e-sports Athlete Points System* (Trial; All-China Sports Federation, 2018; hereinafter referred to as the Points Measures). These documents marked the normative construction of the professional identity of e-sports athletes, granting it legal legitimacy.

The interview data indicate that the formation of professional identity is not simply equivalent to “playing well,” but rather depends on a series of institutionalized procedures, including trials, contracts, registration, tournament qualifications, and club management. When reflecting on their transition from amateur players into the professional system, some respondents emphasized the importance of organizational recognition for the transformation of identity. A current mobile MOBA player stated:

“Previously, I thought that as long as I played well and could make it onto a team, I would be considered a professional player. Later, after actually signing a contract, I realized that professional identity is not that simple. It requires contracts, registration, tournament eligibility, and so on. The club will tell you which tournaments you can participate in and which you cannot. The industry also has corresponding registration and certification requirements. Only after all these procedures are completed will your performance, points, and tournament eligibility be recognized. That was when I felt that being a professional player is not something you declare yourself, but something that must be confirmed through rules and the industry system.” (A3, age 20, Honor of Kings, current professional e-sports athlete).

The development of tournament systems and the increased social visibility of e-sports have further strengthened athletes’ identification with their professional status. Fixed competition schedules, club systems, athlete transfer mechanisms, prize structures, and platform-based livestreaming have gradually led the public to recognize e-sports as a formal competitive profession. A retired player of Honor of Kings reflected on his professional experience as follows:

“When I first started playing, my family and many people around me thought it was just playing mobile games and hardly a profession. Later, as tournaments became more formalized—with fixed schedules, clubs, player transfers, prize pools, and many viewers watching livestreams—people gradually realized that this was not just ordinary gaming. Especially when I played in major tournaments, I felt that I was no longer just proving that ‘I am good at games.’ Instead, I was representing my team in an official competition. That was when my sense of identity as a professional player became stronger, and it also became easier for outsiders to view us as true athletes or professional players.” (A13, age 23, Honor of Kings, retired professional e-sports athlete).

This transformation serves as a concrete manifestation in the digital age of the principle, described by Elias, that deepening networks of dependency lead to the internalization of external constraints. The identity recognition of e-sports players is essentially a process of moving from passive compliance to actively internalizing the norms of the interdependent network as the core of their identity. Furthermore, this process reveals the generative logic of new occupations in the digital age: the rigid framework of rules provides a foundation of legitimacy, while the flexible innovation of market practices expands the meaning of professional identity. Their dialectical interaction not only enables the professional identity of e-sports players to gain legal and social recognition but also demonstrates the dynamic tension between individual autonomy and the evolution of competitive forms within the civilizing process.

### 3.2 Rationalization of bodily discipline: from “free operation” to “data control”

In his analysis of the courtization of knights, Elias pointed out that “the core of bodily discipline is the transformation of the natural body, as an instrument of violence, into a professional body adapted to the demands of complex social cooperation. This process follows the logic of a gradual transformation from external coercion to self-constraint.” The civilizing evolution of the e-sports athlete’s body is an extension of this logic into the digital competitive arena. Using datafication as the core medium, this process drives the body through a civilizing progression from the natural body to the data-disciplined body and ultimately to the solidified professional body. The body gradually becomes the object of competitive capital, technical rationality, and industry rules, with the intensity and refinement of discipline continuously deepening as the professionalization process advances, forming an isomorphic evolution with the institutionalization of identity.

#### 3.2.1 Quantitative transformation of training time: from interest-driven elasticity to regulated rigidity

“The discipline of time is the primary hallmark of civilization.” When medieval knights shifted from random warfare to being on call according to the court schedule, time transformed from a natural rhythm into a social rhythm. The same principle applies to the discipline of training time for e-sports athletes, and it must be delineated according to the stage-specific characteristics of time domestication.

In the pre-professionalization stage of e-sports, the training time of players exhibited a distinct characteristic of “interest-driven elasticity.” The duration of practice was entirely subject to biological instincts and fluctuations in interest, with the start and end points determined by the natural benchmarks of physical fatigue thresholds and the saturation point of pleasurable experience. At this stage, the body served merely as a concrete carrier of natural time and had not yet been incorporated into the rigorous framework of social discipline. For instance, in the early competitive communities of games such as Quake and Counter-Strike in the early 2000s, players primarily practiced in LAN party or internet cafe settings, flexibly adjusting training duration based on fluctuations in interest, without any institutionalized planning (Weiss, 2015).

The development of the professional e-sports system transformed this traditionally loose and relatively unstructured training model. Training time gradually shifted from flexible arrangements to precise regulation. Through fixed daily schedules, collective training, practice matches, tactical discussions, and post-match reviews, clubs divided previously continuous and loosely organized gaming time into distinct training modules. Training was no longer simply a matter of increasing the number of hours spent playing; instead, it was planned, allocated, and supervised according to team objectives. Basic mechanical training, tactical coordination, practice matches, and match reviews each performed different functions and together constituted the daily work routine of professional e-sports athletes. This temporal arrangement fundamentally changed the relationship between athletes and training (Białecki et al., 2024; Carr, 2007). In response, a current player of a mobile MOBA game noted during the interview:

“After joining a professional team, the most noticeable change is that your time is scheduled in great detail. Before, I could play whenever I wanted. Now, when to get up, when to train, and when to do match reviews are largely fixed daily. After training matches, it’s not just about playing. The coach will look at the data, checking whether my laning phase was problematic, whether my rotations were slow, and whether my team-fight execution was correct. Even if I am very tired, I still have to train with the team at the scheduled time, because this is part of the team’s overall rhythm. You cannot just stop whenever you feel like resting.” (A1, age 22, Honor of Kings, current professional e-sports athlete).

Within the professional training system, temporal discipline is manifested not merely in an increase in training hours, but more fundamentally in the decomposition of training content into a set of fixed modules, requiring players to complete daily tasks according to a predetermined schedule. A player of a PC-based MOBA game noted:

“After joining the team, gaming is no longer a leisure activity you engage in only when you feel like it. There are fixed training modules every day: basic mechanics, practice matches, and match replay reviews all need to be completed according to plan. Even when physically tired, you cannot simply stop, because the training volume is set in advance, and neglecting any part would affect subsequent team coordination.” (A8, age 22, DOTA2, current professional e-sports athlete).

The transition from unstructured practice to standardized training is characterized by the gradual sedimentation of external time arrangements into the player’s internal bodily order. After entering the professional sphere, players can no longer determine their training rhythm based on personal preference or physical and emotional states. The daily cycle of training plans, post-match reviews, and team coordination requirements gradually shapes professional habits characterized by regular routines, focused engagement, and active mitigation of fatigue. Training time ceases to be merely a managerial tool and becomes an internal basis for self-regulation. Simultaneously, the physical form and behavioral logic of the e-sports athlete undergo a complete transformation. The playing body, once oriented toward entertainment, is converted into a professional body capable of enduring high-intensity, routinized training. This fully illustrates the evolutionary pathway of the civilizing process, in which external discipline gradually transforms into bodily self-discipline.

#### 3.2.2 Evolution of operational metrics: from natural sensation to data calibration

In the symbol theory, Elias proposed that a key feature of the civilizing process is the gradual transformation of non-essential elements (such as courtly etiquette or, in this context, data) into the new essence of the body. The “data calibration” of e-sports operations is a clear manifestation of this principle (Elias, 1991).

In the pre-professionalization stage, players’ operations relied on natural sensation. Finger click frequency and mouse movement amplitude were entirely based on the body’s intuitive response to the game situation. Evaluation criteria focused on the final score, and innate bodily attributes such as reaction speed and hand-eye coordination were the core of competitive ability. This aligns with Elias’s description of the primitive state of the body before social disciplining.

The “data calibration” of the professionalization stage has fundamentally reshaped the underlying logic of e-sports operations. Metrics such as skill accuracy, rotation time, death location, team-fight participation, and actions per minute made previously tacit aspects of performance visible and comparable. Through data-based reviews, athletes were expected to identify inefficiencies and adjust their movements, decisions, and tactical choices accordingly. A current player of a mobile MOBA game commented:

“After joining the team, many things can no longer be judged solely by feel. Previously, I thought I had played a game decently, but during reviews, the coach would pull up the data—skill accuracy, rotation time, death location, and whether abilities were used too early in team fights. Later, I realized that some actions that felt smooth to me were actually very inefficient in terms of data. As training progressed, I began to think first about whether an action was optimal, rather than relying only on how it felt at the moment.” (A6, age 23, Honor of Kings, current professional e-sports athlete).

In the professional development of the StarCraft series, players’ APM underwent significant technical evolution, rising from an average of 200 per minute in the early days to over 400 per minute among top modern players. As competitive levels increased, relying solely on APM became insufficient to accurately measure operational efficiency, making EPM a more critical evaluation dimension. The EPM metric, defined explicitly as “non-spam actions,” systematically excludes meaningless redundant clicks and invalid commands from APM, thereby providing a more authentic and precise reflection of a player’s practical operational quality and competitive decision-making efficiency. This indicator effectively identifies “empty operations” and promotes the scientific evaluation of e-sports players’ skill levels (Lewis et al., 2011). Data functions not only as an external tool of evaluation but also as a framework through which athletes come to understand their own performance. At first, coaches and analysts use data to identify problems. Over time, however, athletes begin to anticipate these standards and monitor their own actions during training and competition. Evaluation therefore shifts from external correction to continuous self-calibration.

The outcome of this domestication is the marginalization of bodily intuition. Players gradually develop a behavioral pattern in which data feedback takes precedence over tactile feel. The body thus transforms from the subject of the competitive experience into a terminal for data execution, echoing Elias’s civilizing logic that social norms reconstruct the very essence of the body. In this context, e-sports data functions like courtly etiquette: both are non-essential elements that, by virtue of the demands of the social structure, become the core determinants defining bodily behavior.

#### 3.2.3 The deeper shift in disciplinary logic: from bodily autonomy to competitive alienation

Civilization harbors internal contradictions. When discipline serves efficiency to an excessive degree, it can become alienated into a “shackle on bodily subjectivity.” The competitive alienation of bodily discipline among e-sports athletes is a manifestation of this contradiction. In the professionalization process of e-sports, the underlying logic of bodily discipline has shifted from the natural regulation of bodily autonomy to the systematic control of competitive alienation. The synergy of capital and technology molds the digitized body into an appendage of competitive efficiency, and the body’s subjectivity is gradually dissolved under the goal of maximizing competitive performance. This deep transformation is not only an inevitable outcome of the aforementioned mechanisms of time quantification and operational indicatorization but also a typical manifestation of instrumental rationality overriding value rationality in modern competitive sports.

In the pre-professionalization stage, bodily autonomy manifested as the body’s primary control over competitive behavior. Training duration was dynamically adjusted according to biological fatigue thresholds, and operational precision was naturally calibrated through tactile intuition. Discipline at this stage stemmed only from the basic constraints of game rules, and the body always remained the subject, rather than the object, of the competitive experience. For example, in the operational logic of early e-sports players, mouse movement amplitude and keystroke frequency served solely the immediate goal of defeating opponents, rather than preset digital metrics. The body’s natural responses and competitive demands formed a primitive dynamic balance.

During the professionalization process, competitive alienation reconstructed this balance through the dual intervention of capital and technology. Capital logic transformed the aforementioned time quantification into a regulatory tool for competitive labor. The institutionalized training duration of dozens of hours per day essentially breaks down bodily time into measurable units of “competitive output.” Through the linear presupposition of duration, biological time is forced to submit to capital’s pursuit of “maximizing competitive value.” Technological logic, meanwhile, escalates operational indicatorization into “data tyranny.” Metrics such as APM and EPM are not only evaluation criteria but also rigid commands shaping bodily behavior. When the “optimal operational path” is revered as the ultimate standard, and when players must adjust muscle memory according to dozens of detailed metrics, the body degenerates into a “digital terminal” executing algorithmic will, with its original perceptions suppressed as impediments to efficiency.

The alienating characteristics of this competitive disciplinary logic are evident in three dimensions: first, alienation between body and self. Players’ perception of their own bodies is no longer based on internal sensation but anchored to the external feedback of data dashboards. When the system indicates delayed skill release, even if bodily intuition judges the operation to be correct, players are still forced to adjust their movement patterns, resulting in a cognitive reset where data takes precedence over the self. Second, alienation between body and labor. Training behavior shifts from interest-driven activity to the mere fulfillment of metrics. For example, in League of Legends competition, to improve last-hitting success rates, players may need to repeat thousands of mechanical operations until their bodies form conditioned reflexes detached from the game context. The creativity and pleasure of labor are replaced by the mechanical nature of data optimization. Third, alienation between body and health. The rigidity of both time discipline and operational indicators jointly fosters competitive overexertion. Physical and mental health issues among professional players—such as repetitive strain injuries, cervical spine problems, and competitive anxiety—have become industry norms. Narratives that sacrifice health balance for data metrics are even sometimes framed as professionalism, illustrating the impact of competitive disciplinary logic on bodily authenticity. A retired player of a mobile MOBA game reflected on his professional training experience:

“In a professional team, it’s often not about whether you feel you are in good form; it’s about the data. Things like last-hitting, team fight participation, skill accuracy, and rotation time—if any of these are insufficient, they will be brought up during reviews. Sometimes your wrist is already very sore and your eyes are very tired, but if the data hasn’t met the requirements, you still have to keep practicing. After a while, you get a feeling that your body is not your own, but rather that you constantly have to adapt to those metrics.” (A14, age 23, Honor of Kings, retired professional e-sports athlete).

However, the application of artificial intelligence technologies in e-sports training scenarios offers new possibilities for alleviating this alienation dilemma. AI training systems, through precise calculation of training load, biomechanical correction of operational posture, and real-time monitoring and intervention of competitive psychological states, can support players’ health maintenance and scientific training while ensuring training efficiency and the achievement of data metrics. This can, in turn, establish a certain degree of balance and buffer between competitive goals and bodily authenticity.

The pre-professionalization stage of bodily autonomy preserved the integrity of the body as the subject of experience. In contrast, the competitive alienation of professionalization, through capital’s segmentation of time and technology’s monopolization of data, reconstructs the body as a mere vehicle for competitive efficiency. This alienation is not an accidental feature of civilization, but rather a paradox of a civilizing process that prioritizes efficiency. While discipline enhances competitive performance, it may also undermine bodily subjectivity. The future development of e-sports professionalization must return to the civilizing essence of balancing social demands with individual health, preventing bodily discipline from becoming alienated into a new form of physical shackle in the relentless pursuit of efficiency maximization.

### 3.3 Internalization of emotional management: the civilization of emotions from “instinctive release” to “disciplined restraint”

Elias pointed out that “the refinement of emotional control is a core hallmark of civilization,” driven by the deepening of interdependence, fear, and the restraint of emotions. The emotional management of e-sports athletes is an expression of this logic in the digital age. The internalization of emotional management forms an isomorphic civilizing evolution with the institutionalization of identity and bodily discipline discussed earlier. Moreover, using digital technology as a medium, it achieves the domestication from external technical intervention to self-directed emotional restraint, representing both an extension and an innovation of Elias’s theory in the virtual competitive arena.

#### 3.3.1 Pre-professional stage: institutionalized deviance in emotional expression

During the pre-professionalization to nascent professionalization stage, the emotional expression of e-sports players was characterized by emotional catharsis entirely dependent on immediate feedback from competitive outcomes, lacking systematic constraints. This was essentially a natural state where professional ethics had not yet intervened in the emotional domain. Emotional release was completely governed by the immediate feedback of winning or losing, with no institutionalized management system yet established. The player’s identity had not yet transformed into a professional identity, and their emotional expression was essentially an instinctive reaction to frustration, often released through primitive means such as physical violence and verbal aggression. There was no conscious awareness of emotional management, nor systematic recognition of the impact on teams, tournaments, or the industry as a whole.

In early e-sports events, players losing emotional control due to technical malfunctions or disputed rulings was not merely a matter of individual psychological issues, but rather a manifestation of structural anomie. As Zhou Xueguang pointed out, institutional voids and lack of authority can lead individuals to express dissatisfaction through extreme means (Zhou, 2017). Moreau et al. further noted that technical uncertainty and rule opacity systematically trigger players’ anger responses (Moreau et al., 2024). In early LAN tournaments for Counter-Strike and StarCraft, incidents of players smashing keyboards, verbally abusing opponents, or even attacking referees due to disputed rulings or technical malfunctions were documented. Their emotional logic followed biological instincts entirely: wild joy at victory, explosive anger at defeat, with little buffer in between.

This state shares commonalities with the emotional expression of athletes during the professionalization transition stage in traditional sports. During the professionalization of tennis in the 1950s and 1960s, players frequently expressed dissatisfaction with rulings in emotional ways (Abramitzky et al., 2012). American tennis legend Pancho Gonzalez, known for frequent arguments with umpires during this period, repeatedly exhibited loss of emotional control in professional tour matches due to disputed line calls. At that time, professional tennis tournaments lacked uniform disciplinary norms. Gonzalez, as a representative of early professional players, often engaged in heated disputes with umpires over rulings. His volatile on-court image was a microcosm of the institutional deficiencies during early professionalization, a phenomenon directly related to the lack of standardized management during tennis’s transition from amateurism to professionalism.

However, emotional catharsis in e-sports was more prone to boundary violations due to the spatial ambiguity of virtual competition. e-sports players’ verbal abuse was directed not only at opponents and referees but also spread to the surrounding audience through LAN connectivity, creating an emotional contagion effect. Nevertheless, the commonality between the two is evident: both stemmed from the lack of systematic institutional management and the absence of a defined professional identity, leading to a lack of emotional responsibility. Emotional restraint was not yet considered a core dimension of professionalism. Their emotional expression belongs to what Elias described as “primitive impulses not yet socially disciplined.”

#### 3.3.2 Institutionalization stage: the internalization of emotional management among e-sports athletes

After the 21st century, players became dependent on virtual network resources. Any loss of emotional control—such as smashing equipment or verbally abusing opponents—could lead to a rupture in these dependencies, including loss of audience, sponsor withdrawal of funding, and suspension from competition. This fear became the driving force behind emotional restraint. Concurrently, digital technology transformed emotional control from a vague set of rules into a precise intervention system, creating an institutional regulatory framework of monitoring and intervention.

(1) Reconfiguration of Resource Dependence: The Sociogenetic Foundation of Emotional Control.

Elias emphasized that “civilized patterns of behavior are always closely linked to an individual’s function within the social division of labor network and their mode of resource acquisition.” The core transformation in the competitive arena of the 21st century lies in the shift in players’ survival logic from dependence on physical resources to dependence on virtual network resources. Key resources such as audience traffic, sponsor funding, and tournament qualifications are all accumulated and maintained through virtual network platforms, forming a “capital-league-club-network-player-fan” figuration. This structure has fundamentally reshaped the sociogenetic context of emotional control. In traditional sports, loss of emotional control—such as smashing a racket or verbal altercations—primarily affects the immediate outcome of the match. In the virtual network context, however, any act of emotional dyscontrol can rapidly spread through digital media, directly leading to ruptures in dependencies: first, the loss of audience due to a negative image erodes the player’s traffic foundation; second, sponsors withdraw funding based on brand risk, cutting off financial resources; third, tournament organizers invoke rules to impose suspensions, revoking competitive eligibility. The interview data also reveal that professional e-sports athletes’ understanding of emotional dyscontrol is no longer limited to the level of individual personality or immediate reactions, but is directly linked to club reputation, commercial partnerships, and tournament eligibility. A current player of a mobile MOBA game commented:

“When I used to play ranked matches on my own, if I lost and cursed a bit or slammed the keyboard, the worst consequence was that I felt bad. But after turning professional, it’s different. One word, one expression, one impulsive act of yours can be recorded and screenshotted. Fans will see it, the club will intervene, and sponsors will also care. Especially during live broadcasts, if you lose emotional control, it’s not just about losing face for yourself—it affects the team’s image. In serious cases, you might be penalized or even lose the chance to play. So later, I naturally remind myself not to let my emotions come out first.” (A4, age 23, Honor of Kings, current professional e-sports athlete).

(2) Transformation of Value Identification: The Psychogenetic Process of Emotional Internalization.

Elias emphasizes that the internalization of emotional control is a socialization process through which societal prohibitions are transformed into individual self-restraint. In the institutionalization stage of e-sports, the core driving force of this internalization is not traditional moral condemnation, but the real anxiety over the rupture of resource dependencies. When players realize that the direct consequence of emotional dyscontrol is the collapse of their survival foundation, external institutional sanctions become internalized as psychological early warnings. Proactively adjusting mindset before competitions, restraining impulses after defeats, and maintaining a rational image during live streams gradually shift from being passive choices to avoid punishment to active needs for survival security.

This psychogenetic process can be divided into two levels. First, there is the immediate association between consequence anticipation and behavioral restraint. The memorial nature of digital media has made past cases of emotional dyscontrol public knowledge. Furthermore, emotion recognition technology further transforms vague emotions into monitorable and quantifiable data, reinforcing players’ restraint reflexes. For example, e-sports voice emotion recognition models can identify negative emotions such as anger and frustration in real-time by analyzing changes in tone and speech rate (Hung and Chen, 2022). Facial expression emotion recognition models, trained on 2,400 expression images, can capture micro-expressions such as frowning after mistakes or downturned mouth corners after losses, inferring the intensity of emotions. When players experience negative emotions, not only do they automatically associate them with negative precedents, but they can also intuitively perceive emotional risks through the quantitative feedback of technology, forming conditioned reflexes from data warnings to emotional management (Hung et al., 2020). Constrained by tournament league monitoring and punitive measures, along with the structure of resource dependence, players develop a clear understanding of the consequences of emotional dyscontrol and proactively initiate emotional management behaviors, constituting the primary psychological foundation for emotional internalization. A current player of an FPS game commented on emotional control during competitions:

“When you get killed repeatedly by the opponent, or when team communication is not on point, emotions definitely come up. When I used to play on my own, I might have just cursed outright. But after turning professional, I don’t dare to do that. Because team voice chat might be recorded, and people are watching the matches. If emotions show too clearly, the coach will criticize you, and in serious cases, it might even affect your future playing opportunities. So, many times it’s not that I am not really angry, but that I know I cannot show it. I have to hold it in and keep the words to myself, at least not explode during the match.”(A9, aged 23, CS: GO, current professional e-sports athlete).

Second, there is the high-level transformation of value identification and emotional internalization. When technology-assisted emotional restraint becomes a long-term behavioral habit, players gradually incorporate it into their value cognition system of professional identity. That is, a rational and restrained emotional state is regarded as a core hallmark of e-sports professionalism. At this point, emotional control gradually begins to be internalized as part of professional identity, driving its shift from being externally driven by constraints to being a conscious aspect of professional identity. A retired mobile MOBA player reflected on his professional experience:

“When I first started playing professionally, I still felt like complaining when emotions ran high, especially after losing matches or being criticized by fans. But over time, I gradually came to realize that as a professional player, I cannot just focus on my immediate feelings. Many things said, many reactions shown, can affect the team and future matches. Later on, without needing constant reminders from the coach, I would stop myself, think about whether I should say something, whether I should show that emotion. It’s not simply about fearing punishment, but rather that, as a professional player, you should be able to control your emotions. That is also part of professional ability.” (A15, age 23, Honor of Kings, retired professional e-sports athlete).

From a practical perspective, players begin to actively practice the internalization of emotional management through individual coping strategies and team assistance. For example, individual coping focuses on e-sports players using internal strategies to regulate their emotions and maintain psychological well-being, including six emotion regulation strategies: situation selection (actively approaching positive situations or avoiding negative ones), situation modification (adjusting the current emotion-triggering situation), attentional deployment (shifting focus from negative aspects to small in-game goals), cognitive change (reinterpreting events with a positive perspective), response modulation (controlling the outward expression of emotions), and social interaction (seeking emotional support from the community; Kou and Gui, 2020). Regarding specific methods of emotional regulation, respondents also noted that emotional management in professional settings does not simply mean suppressing emotions, but rather achieving self-regulation through a series of practical techniques. A current Honor of Kings player commented:

“When I’m feeling upset now, I usually don’t speak immediately. I first refocus my attention on the next wave of minions or the next team fight. During reviews, if I’m feeling frustrated, I listen to the coach first and then communicate separately with teammates later. Sometimes teammates also remind me not to rush and to stabilize the pace. After playing professionally for a while, you come to understand that it’s not that you cannot have emotions, but that you cannot let them affect your judgment and coordination.” (A6, age 23, Honor of Kings, current professional e-sports athlete).

At the team technical level, players utilize neurofeedback training frameworks combined with their psychological cognitive profiles. Using real-time electroencephalography (EEG) data to analyze the correlation between brain activity and performance indicators such as stress and concentration, they are guided to adapt to optimal brain activity patterns that maintain a dynamic balance between focus and relaxation (Soler-Dominguez et al., 2021). A typical example is the use of stress biofeedback devices in FIFA e-sports scenarios to precisely capture emotional peaks during high-pressure nodes, such as close to scoring or in the tense final moments of a close match. Players adjust their breathing rhythm in real-time based on physiological data feedback such as heart rate and skin conductance, helping them achieve active emotional regulation (Drijfhout, 2020). This transformation from risk-avoidance-driven to value-internalization-driven behavior is a typical manifestation of the synchronous change between sociogenesis and psychogenesis identified by Elias. The structure of resource dependence at the societal level ultimately shapes the structure of emotional cognition at the individual level.

#### 3.3.3 The essence of emotional management internalization: three layers of logic in the professionalization of e-sports athletes

The internalization of emotional management among e-sports athletes—transforming from “instinctive release” to “disciplined restraint”—represents a specific manifestation of Elias’s “refinement of emotional control” in the digital age. Its essence lies in the process by which individuals, within the virtual competitive arena, achieve the rationalization of emotions through digital tools, thereby completing professional socialization. This process operates on three core layers of logic.

First, at the responsibility level, emotional management is deeply bound to digital professional identity responsibility, moving beyond individual cultivation. Due to the virtual resource dependency structure of “capital-league-club-network-player-fan,” digital media amplifies athletes’ emotional expression into a “public text.” Emotional stability directly sustains survival resources such as audience traffic, sponsor funding, and tournament qualifications. Consequently, emotional restraint is elevated from a matter of personal conduct to a professional responsibility for maintaining the resource chain, rather than being merely a rule-based constraint.

Second, at the discipline level, the vehicle for emotional discipline has shifted from the physical rules of traditional sports to self-discipline empowered by digital tools. Tools such as biometric monitoring, neurofeedback training, and emotion recognition-based match reviews transform abstract emotions into measurable, trainable professional indicators. Players can intuitively perceive emotional risks through data and actively regulate themselves, achieving internalization far more efficiently than through external supervision.

Third, at the synergy level, emotional management, identity recognition, and bodily discipline form a resonant paradigm through digital carriers. The institutionalization of identity recognition sets goals for emotional management; bodily discipline establishes the behavioral foundation for emotional management; and the internalization of emotions, in turn, reinforces the deepening of identity recognition and bodily discipline. This coupling of social structure, technological tools, and individual psychology not only expands the boundaries of Elias’s theory but also reveals new pathways for professional civilization in the digital age, providing both a theoretical and practical model for e-sports professionalization. A current mobile MOBA player reflected:

“Looking back now, I think the biggest change from turning professional is not just that my skills improved, but that my entire self has to be framed by professional requirements. When I used to play games as an amateur, I could get frustrated when I lost and say whatever I wanted. But professional players are different: letting emotions get out of control during a match affects your teammates; saying one wrong word during a livestream can be screenshotted by fans; in serious cases, it can affect the club and future playing opportunities. Over time, I came to understand that controlling emotions is not simply about being afraid of punishment, but rather that this is what the identity demands of you. Whether you can stabilize your emotions and continue to play well in a match is actually a major difference between professional players and ordinary gamers.” (A11, age 21, Honor of Kings, current professional e-sports athlete).

The coupling of social structure, technological tools, and individual psychology expands the boundaries of Elias’s theory and reveals new pathways for professional civilization in the digital age, providing both a theoretical and practical model for e-sports professionalization.

### 3.4 Structural tensions in the formation of professional identity: the dialectical duality of the civilizing process and alienation

Previous sections have examined the localized labor alienation generated by training mechanisms from the perspective of bodily discipline. Integrating the three dimensions of professionalization—identity institutionalization, bodily rationalization, and emotional internalization—it becomes evident that the internal contradictions within the e-sports field are not confined to training practices but are deeply embedded in the broader industrial system. Drawing on Elias’s process sociology and his theory of civilization, the professionalization of e-sports demonstrates a dual dialectical character. On the one hand, industry regulations and organizational constraints continuously shape standardized competitive practices, stabilize professional identities, and enhance athletes’ capacity for emotional self-regulation. On the other hand, the intertwined forces of commercial capital, quantitative evaluation systems, and digital platform power construct new forms of hierarchical discipline, generating multilayered forms of labor alienation.

First, professionalization fundamentally reconstructs the relationship between individuals and gaming activities. Ordinary players are primarily motivated by personal interest and entertainment, maintaining considerable autonomy over their gaming time and forms of participation. However, once entering the professional system, players’ gaming practices become incorporated into an integrated institutional framework consisting of club-based training, competitive evaluation, and data-driven assessment. Their operations and match performances are increasingly oriented toward the achievement of competitive outcomes. Consequently, gaming, originally characterized as a leisure activity based on enjoyment and voluntary engagement, undergoes a fundamental transformation in its value orientation and becomes a form of professional labor centered on competitive production.

Second, commercialization reshapes the multifaceted identity of e-sports players. Within the broader e-sports ecosystem, players are not only competitive actors on the battlefield but also commercial entities carrying traffic value and brand interests. Competitive performance, everyday behaviors, livestreaming activities, and interactions with fans are directly connected to club operations and commercial revenues. As a result, players are required not only to continuously improve their competitive abilities but also to manage their public image and emotional expressions. Their physical conditions, emotional characteristics, and individual expressions are gradually transformed into professional resources that can be evaluated, developed, and utilized by commercial actors.

Third, while professional identity provides practitioners with developmental opportunities and social recognition, it simultaneously constrains individual autonomy. Players’ professional qualifications and economic stability are highly dependent on club-based management systems. Standardized training schedules and rigid performance indicators significantly restrict players’ autonomy in allocating time and selecting operational approaches. Therefore, players exist simultaneously as active agents who pursue competitive improvement and as subjects continuously shaped and disciplined by organizational institutions. This creates an enduring internal tension between individual autonomy and institutional demands within the e-sports industry.

Furthermore, e-sports has developed a distinctive governance structure that differs from traditional sports. Compared with conventional sports, which generally possess relatively mature and independent governing organizations responsible for regulating competition and protecting athletes’ interests, professional e-sports remains highly dependent on game developers. Developers not only control game design, rule systems, version updates, and balance adjustments but also influence the direction of professional ecosystems through technological mechanisms and competitive environments. Changes in game versions can directly reshape established tactical systems and training patterns, forcing players to continuously adjust their competitive strategies to adapt to platform rules. Meanwhile, key resources such as tournament operations and livestreaming rights are largely controlled by platforms. Beyond hierarchical management from leagues and clubs, players must also comply with technical standards and commercial rules established by developers, thereby forming a distinctive platform-dependent power relationship.

In conclusion, the construction of e-sports players’ professional identities simultaneously follows two intertwined trajectories: civilizing development and labor alienation. Institutional constraints guide players toward stable and standardized professional behaviors, continuously promoting the professionalization of e-sports. At the same time, commercial interests, quantitative evaluation mechanisms, and platform governance collectively intensify the regulation of players’ time, bodies, and emotions. From the perspective of Elias’s theory of civilization, the civilizing process is not a linear trajectory of progressive development; rather, it is a dynamic process through which power relations and individual behavioral patterns are continuously reconstructed within networks of social interdependence. Therefore, the sustainable development of e-sports requires the establishment of a dynamic balance among competitive achievement, commercial interests, and players’ individual development, preventing professional norms from ultimately becoming rigid constraints that suppress individual agency.

## 4 Extensions and reflections on the theory of the civilizing process

Examining the psychogenesis of emotional internalization in the e-sports field of the 21st century through the lens of Elias’s theory of the civilizing process reveals characteristics distinct from traditional civilizing processes. These characteristics not only enrich the contemporary implications of Elias’s theory but also provoke new reflections.

At the theoretical field level, Elias’s theory of the civilizing process is rooted in physical industrial society, where the internalization of external constraints was long confined to physical interactions in physical space. The virtual nature of the e-sports scene in the digital age has sparked debate about whether civilization is disrupted. However, the civilization of e-sports still follows the logic of social dependency networks, external constraints, and individual internalization, with the vehicle of discipline shifting from physical rules to digital tools. This finding challenges the perception of the theory’s exclusive applicability to physical fields, demonstrating its cross-field adaptability to virtual society. It adds a perspective for studying civilization in virtual fields and provides a new analytical lens for examining virtual community order and digital professional ethics.

At the interdependency network level, Elias emphasizes that “the essence of civilization is the deepening of interdependency networks.” In traditional settings, the driving force for individual emotional control stems from responses to physical dependencies. e-sports, however, extends this network from physical entities to digital actors, shifting dependency ties from face-to-face interaction to digital media connections, and transforming dependency feedback from delayed evaluation to real-time data responses. This extension not only enriches the theoretical scope of social dependency networks but also constructs a new paradigm for the socialization of individuals in digital professions. The essence of individual socialization in the digital age is a response to responsibilities within virtual dependency networks. This logic can be transferred to other digital professions such as virtual streamers and remote programmers, providing theoretical support for understanding the sources of individual responsibility in digital civilization.

At the micro-operational level, current research on digital civilization often focuses on macro-structures or meso-level platform governance, creating a gap between macro-theory and micro-practice. The process of emotional internalization among e-sports athletes provides a crucial sample to bridge this gap. Through a process of monitoring emotional signals with digital tools, intervening in behavior with regulation strategies, and ultimately internalizing rational restraint as professional identity cognition, this sequence—monitoring, intervention, internalization—offers a practical pathway for individual self-development within digital civilization. It also effectively bridges the disconnect between macro-level research and micro-level practice, providing a paradigm for connecting individual behavior to macro-order in constructing the civilizational order of digital society, thereby demonstrating unique value for both the contemporary extension of Elias’s theory and research on digital civilization.

## 5 Optimizing the path for professional development of e-sports athletes

The civilizing process is a dynamic balance between structural social constraints and the maintenance of individual subjectivity. The core contradiction in the current professional development of e-sports athletes lies in the imbalance between discipline and subjectivity. Future path optimization must return to the essence of civilization, both continuing the civilizing process of rule internalization and emotional rationalization, while simultaneously addressing alienation through mechanism innovation to achieve human-centered civilization. The optimization pathway is illustrated in Figure 2.

**Figure 2:**
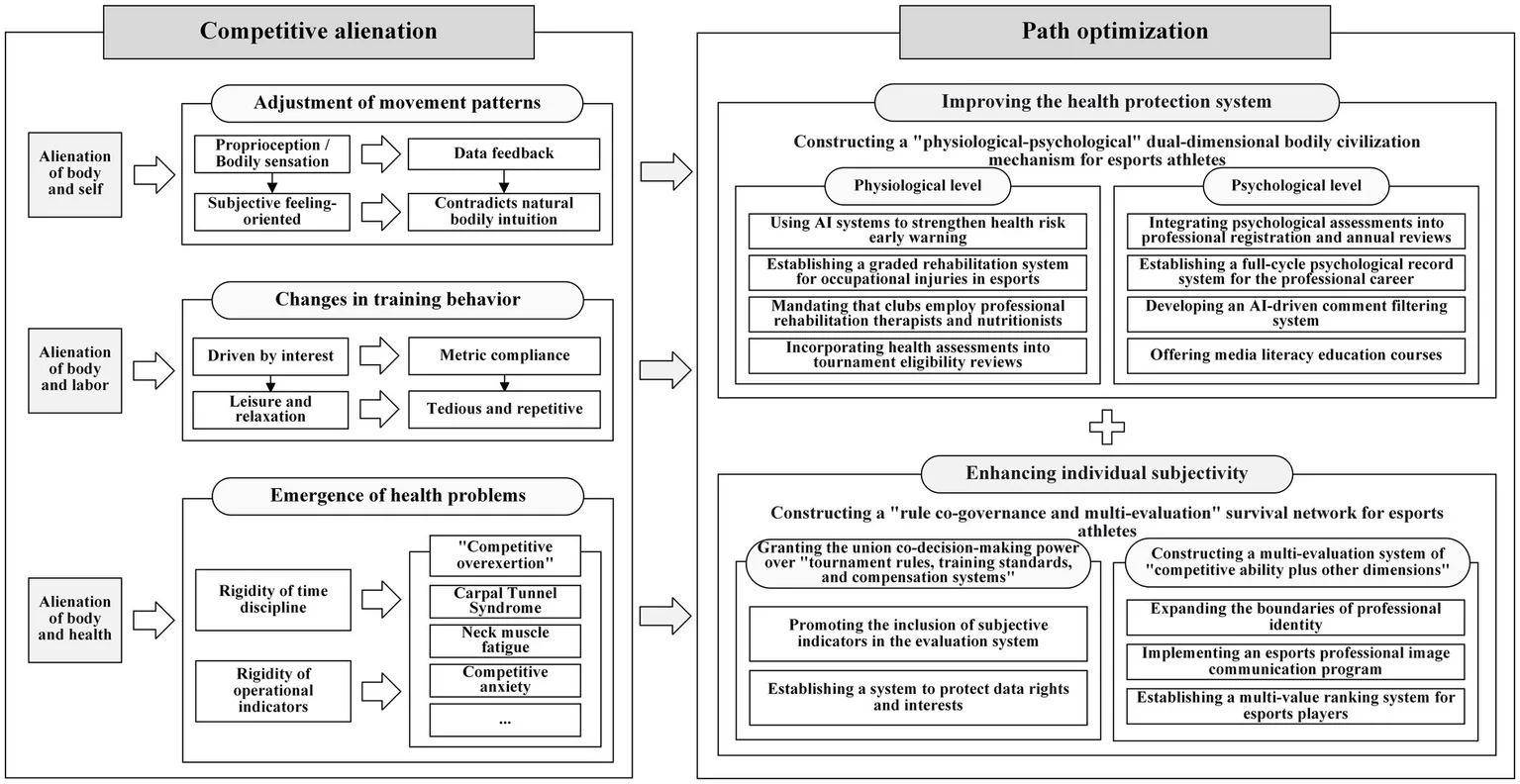
Optimization pathway for the professional development of e-sports athletes.

### 5.1 Improving the health protection system: constructing a “physiological-psychological” dual-dimensional bodily civilization mechanism for e-sports athletes

Elias emphasized that “the goal of bodily discipline is the dynamic coordination of the demands of social cooperation and the survival needs of the individual, not one-sided suppression.” The current bodily discipline of e-sports athletes has fallen into difficulty due to the dominance of capital’s profit-seeking nature and technical instrumental rationality. Issues such as cervical spine strain caused by dozens of hours of daily training and data-prioritization-induced psychological anxiety essentially indicate that bodily civilization has deviated from its sustainable core. In the future, a new mechanism integrating data-driven discipline with health protection should be constructed, centered on the synergy of the physiological and psychological dimensions.

At the physiological level, leveraging the data advantages of e-sports, the indicators of bodily discipline should be expanded from a focus solely on competitive efficiency to include health risk early warning. AI systems can be used to collect real-time data on sitting posture pressure distribution, finger force frequency, heart rate variability (HRV), and other metrics. Health thresholds should be established to trigger automatic interventions. Concurrently, a graded rehabilitation system for occupational injuries in e-sports should be established, mandating clubs to employ professional rehabilitation therapists and nutritionists. Health assessments should be incorporated into tournament eligibility reviews to safeguard athletes’ physical health.

At the psychological level, breaking away from short-term intervention models, a full-cycle psychological record system covering “training - active career - retirement” should be constructed. Psychological assessments should be integrated into professional registration and annual reviews. An AI-driven comment filtering system and media literacy education courses should be developed to prevent acute stress reactions from developing into chronic trauma.

### 5.2 Enhancing individual subjectivity: constructing a “rule co-governance and multi-evaluation” survival network for e-sports athletes

The key to enhancing individual subjectivity in the power dynamic lies in achieving a balanced reconstruction of the interdependency network. Elias’s theory of “interdependency networks” points out that dependencies in the civilizing process are bidirectional interactions, not one-sided attachments. Currently, e-sports athletes find themselves in a unidirectional dependency due to platforms and clubs controlling tournament resources and holding data monopolies, leading to a power imbalance. In the future, the interdependency network should be reconstructed through organizational empowerment and institutional participation.

First, drawing on the experience of traditional professional sports leagues and integrating the globalized and digital characteristics of e-sports, a cross-regional e-sports players’ union should be established. This union should be granted co-decision-making power over “tournament rules, training standards, and compensation systems.” For example, it could negotiate a maximum daily training duration not exceeding a specified number of hours, including physical training time, and promote the inclusion of subjective indicators in evaluation criteria. At the same time, regulations should clearly establish the co-ownership of training data and match recordings, prohibiting clubs or platforms from unilaterally using them for commercial development such as AI training or paid match reviews. If such data are to be used, clubs must sign revenue-sharing agreements with players to protect their data rights.

Second, the single evaluation logic prioritizing only competitive performance should be broken. A multi-evaluation system of “competitive ability plus other dimensions” should be constructed, incorporating elements such as tactical analysis, youth training instruction, e-sports science popularization, and anti-cyberbullying advocacy into professional certification. For example, players could expand their professional identity boundaries by obtaining certifications as youth coaches or passing e-sports commentator qualification exams. Industry associations could take the lead in implementing an e-sports professional image communication program, organizing players to participate in youth digital literacy education and anti-cyberbullying themed activities to break the public stereotype that e-sports equates to gaming addiction. A multi-value ranking system for e-sports players should be established, conducting annual awards based on dimensions such as competitive performance, social contribution, and professionalism, enabling players to strengthen their subjectivity through diverse value creation and upgrading the interdependency network from unidirectional resource extraction to bidirectional value symbiosis.

## 6 Conclusion

Grounded in Elias’s theory of the civilizing process, this study systematically investigates how e-sports athletes transition from amateur players to professional competitors, while examining the coexistence of professional advancement and structural alienation within this trajectory. Findings indicate that athletes’ professional transformation is far more than a simple improvement of competitive skills. Instead, it constitutes a prolonged socialization process: embedded within an interdependent network spanning tournament frameworks, club organizations, commercial capital, digital platforms, and fan communities, athletes experience continuous reshaping of their identities, bodies, and emotional regulation. Shaped jointly by institutional rules, data-centered performance metrics, and public normative discourse, amateur gamers progress through three interwoven shifts—institutionalized professional identity, rationalized bodily discipline, and internalized emotional regulation—to emerge as professional agents capable of self-regulation and adherence to occupational standards.

This research further unpacks three interconnected dimensions that constitute the civilizing process of e-sports professionalization. First, tournament structures, official registration protocols, employment contracts, and industry accreditation collectively institutionalize professional identity, transforming casual, self-motivated game participants into formally recognized professional competitors within the e-sports ecosystem. Second, standardized training regimens, quantitative competitive benchmarks, and data review frameworks rationalize bodily practice. Athletes’ motor movements, in-game operational rhythms, and match performances are continuously quantified, refined, and disciplined, transforming the body into a professional resource subject to ongoing management and optimization. Third, competitive pressure, team collaboration norms, platform communication guidelines, and commercial demands transform spontaneous emotional expression into regulated professional conduct; emotional self-management thus becomes a core professional competency. Taken together, e-sports professionalization reflects Elias’s core civilizing logic—the gradual transformation of external constraints into internal self-control—while simultaneously generating structural tensions, including technological surveillance, reduced individual autonomy, and labor alienation.

Theoretically, this study extends Elias’s civilizing process theory from traditional sporting contexts to digital competitive spaces, unpacking the sociogenetic and psychogenetic mechanisms underlying digital professionalization. Unlike traditional sports, which rely primarily on sporting organizations, associations, and competition systems to establish professional regulation, e-sports’ regulatory architecture is deeply intertwined with platform governance, real-time data tracking, and algorithmic assessment. This renders e-sports’ civilizing process characterized by datafication, performance visualization, and technological mediation, thereby expanding the explanatory scope of Elias’s framework within digital professional contexts.

Practically, this study provides implications for e-sports industry governance and athletes’ professional development. The development of the e-sports industry requires a balance between competitive efficiency and athlete well-being by establishing integrated physical and psychological health protection systems to mitigate the physical and mental burdens caused by high-intensity and data-driven training. Meanwhile, the industry should improve collaborative governance mechanisms and multidimensional evaluation systems, expand channels for athlete rights protection, reduce excessive reliance on performance-based and traffic-oriented evaluations, and promote a sustainable model of e-sports professionalization that integrates competitive achievement, professional ethics, physical and mental health, and long-term career development.

This study has several limitations. First, the findings are primarily based on empirical data from Chinese e-sports athletes, and their cross-national, cross-regional applicability requires further validation. Second, although the study includes players from mobile MOBA, PC MOBA, and FPS games, it does not sufficiently examine disciplinary differences across game genres, club governance models, or career stages. Future research should further compare the professionalization mechanisms, disciplinary pathways, and retirement transitions of e-sports athletes across different game genres, and continue to examine the impact of AI training systems, digital media dissemination, and platform governance mechanisms on the career process of e-sports athletes.

## Data Availability

The original contributions presented in the study are included in the article/supplementary material, further inquiries can be directed to the corresponding authors.
